# Mir-195-5p targets Smad7 regulation of the Wnt/β-catenin pathway to promote osteogenic differentiation of vascular smooth muscle cells

**DOI:** 10.1186/s12872-024-03891-2

**Published:** 2024-04-23

**Authors:** Wei Lin, Lianglei Hou, Jialyu Tang, Anwu Huang, Zhuyin Jia

**Affiliations:** 1 Department of Intervention, Wen Zhou Central Hospital, Wenzhou, 325000 China; 2 Department of Intervention, Wen Zhou People’s Hospital, Wenzhou, 325041 China; 3 Panvascular Disease Management Center (PVDMC), Wen Zhou Central Hospital, Wenzhou, 325000 China

**Keywords:** miR-195-5p, Wnt/β-catenin pathway, Osteogenic differentiation, VSMCs, Smad7

## Abstract

In this study, we sought to investigate the mechanisms of action of miR-195-5p in the osteogenic differentiation of vascular smooth muscle cells (VSMCs), and thereby provide novel insights and a reference for the targeted therapy of arterial media calcification. VSMC differentiation was induced using sodium β-glycerophosphate, and we investigated the effects of transfecting cells with miR-195-5p mimics, vectors overexpressing Smad7, and the Wnt/β-catenin pathway inhibitor (KYA1797K) on VSMC differentiation by determining cell viability and apoptosis, and the mRNA and protein expression of factors associated with osteogenic differentiation and the Wnt/β-catenin pathway. The results revealed that miR-195-5p mimics enhanced the osteogenic differentiation of VSMCs induced by β-glycerophosphate, whereas the overexpression of Smad7 reversed this phenomenon. In addition, KYA1797K was found to promote the effects of Smad7 overexpression. In conclusion, by targeting, Smad7, miR-195-5p promotes the Wnt/β-catenin pathway. and thus the osteogenic differentiation of VSMCs. These findings will provide a reference for elucidating the mechanisms whereby miR-195-5p regulates osteogenic differentiation.

**Supplementary Information:**

The online version contains supplementary material available at 10.1186/s12872-024-03891-2.

## Introduction

The osteogenic trans-differentiation of vascular smooth muscle cells (VSMCs) to calcified cells plays a central role in arterial media calcification (AMC) [1], which is an independent predictor of cardiovascular mortality [2]. VSMCs, a major components of blood vessels, regulate physiology [3], are associated with atherosclerosis, neurodegenerative diseases, and vascular diseases [4–6], and are also involved in osteoblast differentiation [7, 8]. The development of AMC is associated with vascular calcification, a biological process similar to bone formation, including osteogenic differentiation [9], and consequently, it is important to determine the mechanisms underlying the successful differentiation of VSMCs.

MicroRNAs (miRNAs) are a class of non-coding RNAs involved in the progression of numerous diseases and disorders; however, few studies have examined the associations between AMC and miRNAs. Among these RNAs, miR-195-5p has been established to be associated with tumor biology [10], gestational diabetes, heart failure, and schizophrenia [11–13], and Chang et al. have also reported that osteoblast differentiation is regulated by miR-195-5p [14]. To date, however, there have been few reports on differentiation mediated by miR-195-5p regulation. Given the wide-ranging regulatory roles of miR-195-5p, it is desirable to gain a more comprehensive understanding of the mechanisms underlying miR-195-5p-mediated regulation.

Osteogenic differentiation has also been established to be regulated by the Wnt/β-catenin pathway [15, 16], with which Smad7 is closely associated [17]. Bao et al. have reported that Smad7 mediates the SOX7 and Axin-2 regulation of the Wnt/β-catenin pathway to influence to progression of breast cancer [18], whereas by targeting Smad7, miR-15b inhibit the osteogenic differentiation of bone mesenchymal stem cells [19]. Similarly, Wei et al. have observed that miR-17-5p and its target gene *SMAD7* regulate the differentiation and proliferation of osteoblasts [20]. Numerous studies have evaluated the associations between miR-195-5p and the Wnt/β-catenin pathway. For example, Chen et al. found that miR-195-5p can regulate the Wnt/β-catenin pathway in patients with renal cell carcinoma [21], whereas Zhu et al. have reported that this miRNA can inhibit activation of the Wnt/β-catenin pathway-mediated regulation of hair follicle induction of dermal papilla cells [22].

To the best of our knowledge, however, it has yet to be determined whether miR-195-5p plays a role in regulating the osteogenic differentiation and calcification of VSMCs. In this regard, we hypothesized that by targeting Smad7, miR-195-5p may contribute to regulating the differentiation of VSMCs to osteoblast-like cells via the Wnt/β-catenin pathway. In this study, based on the osteogenic induction of VSMCs by β-glycerophosphate (β-GP), we examined the expression of miR-195-5p and osteogenic markers before and after osteogenic induction, and assessed the effects of transfection with miR-195-5p mimics and/or an Smad7 overexpression vector (ov-Smad7), and Wnt/β-catenin pathway inhibitors. We believe our findings in this study will provide a basis for further in-depth studies on the mechanisms of action of miR-195-5p in the osteogenic differentiation of VSMCs, as well as novel insights and a reference for the targeted therapy of AMC.

## Materials and methods

### Cell culture

Rat VSMCs, obtained from Wuhan Punosai (Wuhan, China), were cultured in Dulbecco’s modified Eagle’s medium (DMEM: SH30022.01B; Hyclone, USA) and 10% PBS (P1010; Solarbio, China) in a 5% CO_2_ atmosphere at 37 °C.

### Construction of a rat VSMC osteogenic induction model

Model group rat VSMCs were cultured in an incubator at 37 °C in a 5% CO_2_ atmosphere. On reaching 80–90% confluence, the cells were digested with 0.25% trypsin and used to inoculate the wells of six-well plates containing 2 mL of complete medium at a cell density of 1 × 10^5^ cells/well. The cells were incubated for 24 h at 37 °C in a 5% CO_2_ atmosphere, after which, the plates were removed and the complete medium was carefully aspirated from the wells, followed by the addition of 2 mL of osteogenic induction medium containing 10 mM β-GP (D302990; Aladdin, China). The cells were thereafter induced for 0, 3, 7, and 14 d for subsequent assays. Control cells were cultured normally in the absence of osteogenic induction.

### 3-(4,5-Dimethylthiazol-2-yl)-2,5-diphenyltetrazolium bromide (MTT) assay

To evaluate VSMC proliferation, we performed an MTT assay. The cells were cultured in 96-well plates (3 × 10^3^ cells: 167,425; Thermo Fisher Scientific), and following the addition of 10 µL of MTT solution (M1025; Solarbio) were further cultured for 4 h, after which the optical density of well contents was measured at 490 nm using an AMR-100 spectrometer (ALLSHENG, China).

### Flow cytometric analysis

VSMCs (1 × 10^6^) were initially resuspended in culture medium and centrifuged at 400 × *g* and 4℃ for 5 min. The pelleted cells were then resuspended in 200 µL of 0.01 M PBS and stained with 10 µL of Annexin V-fluorescein isothiocyanate (556,547; BD, USA) and 10 µL of propidium iodide in the dark for 30 min. Thereafter, following the addition of 300 µL of 0.01 M PBS, we performed flow cytometry using a NovoCyte flow cytometer (ACEA Biosciences, China).

### Quantitative real-time polymerase chain reaction

Total RNA was extracted from VSMCs using TRIzol reagent (15,596,026; Ambion; Thermo Fisher Scientific, USA) and reverse-transcribed to first-strand cDNA using a reverse transcription kit (6215 A; TaKaRa Biotechnology Co., Ltd., Dalian, China). The cDNA was subsequently subjected to PCR amplification using a SYBR Green PCR Kit (KM4101; KAPA Biosystems; Boston, USA), with the amplification products being analyzed using the 2^−ΔΔCq^ method. The sequences of the primers used for amplification are shown in Table 1.

**Table 1:** The sequences of primers used for quantitative real-time polymerase chain reaction

Gene name	Primer sequences
miR-195-5p	F: GGGGTAGCAGCACAGAAAT
	R: CTGGTGTCGTGGAGTCGG
U6 (internal reference of miRNA)	F: CTCGCTTCGGCAGCACA
	R: AACGCTTCACGAATTTGCGT
Runt	F: CGGGCAATGACGAAAAC
	R: GCTCGGAAAAGGACAAACT
RUNX2	F: GGACGAGGCAAGAGTTTCAC
	R: ACTGGGATGAGGAATGCG
bone morphogenetic protein (BMP) 2	F: CGAGAAAAGCGTCAAGCC
	R: CCACATCACTGAAGTCCACATAC
alkaline phosphatase (ALP)	F: GGTGTCGGAAGATGGGA
	R: CAAAGGAATGTTAGGGGC
osteocalcin (OCN)	F: GGAGGGCAGTAAGGTGGTGAA
	R: GAAGCCAATGTGGTCCGCTA
Smad7	F: GTGCGTGGTGGCATACTGG
	R: CGATCTTGCTCCTCACTTTCTG
GAPDH (internal reference of mRNA)	F: CCACTCCTCCACCTTTG
	R: CACCACCCTGTTGCTGT

### Western blotting

Total proteins were extracted from VSMCs using a radioimmunoprecipitation assay lysis buffer (R0010; Solarbio, China) and quantified using a bicinchoninic acid kit (PC0020; Solarbio, China). Aliquots (20 µg) of the extracted proteins were separated on polyacrylamide gels and transferred onto polyvinylidene fluoride membranes (IPVH00010; Millipore, USA). Having initially blocked the membranes, these were then incubated for 1 h with primary antibodies against RUNX2 (PAB33483), Smad7 (PAB40077), Wnt3a (PAB30170), β-catenin (PAB30715), and GAPDH (PAB36269) at a dilution ratio of 1:1000, all of which were purchased from Bioswamp (Wuhan, China). Thereafter, the membranes were incubated with a goat anti-rabbit IgG secondary antibody (SAB43714; Bioswamp, China) for 1 h.

### Transfection

Prior to transfection, VSMCs (5 × 10^5^) were seeded in the wells of six-well plates for 24 h. Thereafter, 100 pmol miRNA was diluted in 250 µL Opti-MEM (31985-062; Gibco, USA) with thorough mixing, and similarly, 5 µL of Lipofectamine® RNAiMAX (11668-027; Invitrogen, USA) was diluted in 250 µL of Opti-MEM and left to stand for 5 min. The two solutions were then evenly mixed and incubated at 25℃ for 20 min to obtain a complex, 500 µL of which was added to the cells, with subsequent shaking. Following a 4-h incubation, the medium was replaced, and the cells were cultured for a further 24 h, after which the expression of the transferred genes was determined.

### Dual-luciferase reporter assay

Smad7 cDNA containing the predicted binding sites of miR-195-5p was inserted into wild-type (WT) pmirGLO-Smad7 vectors. Similarly, mutant (MT) Smad7, comprising point mutations in the miR-195-5p seed-region binding site, was inserted into MT pmirGLO-Smad7 vectors. VSMCs were co-transfected with Smad7-WT or Smad7-MT plasmids and miR-195-5p mimics or miR-NC for 4 h using Lipofectamine 2000 (Invitrogen). A dual-luciferase reporter assay kit (RG027; Beyotime, Shanghai, China) was used to measure cell luciferase activities accordingly manufacturer’s recommended protocol.

### Alizarin red staining

VSMCs were initially washed with PBS and fixed in 4% paraformaldehyde for 30 min. Having subsequently removed the paraformaldehyde solution, 2 mL alizarin Red staining solution (M040; Shanghai Gefan Biotechnology Co., Ltd., China) was added to the cells, and after 30 min, the cells were washed with distilled water and photographed under an inverted microscope.

### Alkaline phosphatase staining

VSMCs in each group were cultured in six-well plates and washed with 0.01 M PBS, followed by fixing in 2 mL of 4% paraformaldehyde for 15 min, and then further rinsed with 0.01 M PBS. Thereafter, the cells were stained for 15 min with 2 mL of alkaline phosphatase (ALP) staining solution (G1481; Solarbio) shielded from light, and then washed with 0.01 M PBS and photographed under an inverted microscope.

### Statistical analysis

Statistical analyses were performed using SPSS 23.0 software. All data are presented as the means ± standard deviation. Each of these aforementioned assays were performed using three biological replicates. For comparisons between the two groups, we used Student’s *t*-test, and one-way analysis of variance followed by Tukey’s post hoc test was used to compare more than two independent groups. A p-value < 0.05 was considered to be indicative of a statistically significant difference.

## Results

### Osteogenic differentiation of VSMCs is associated with activity of the Wnt/β-catenin pathway

**Fig. 1:**
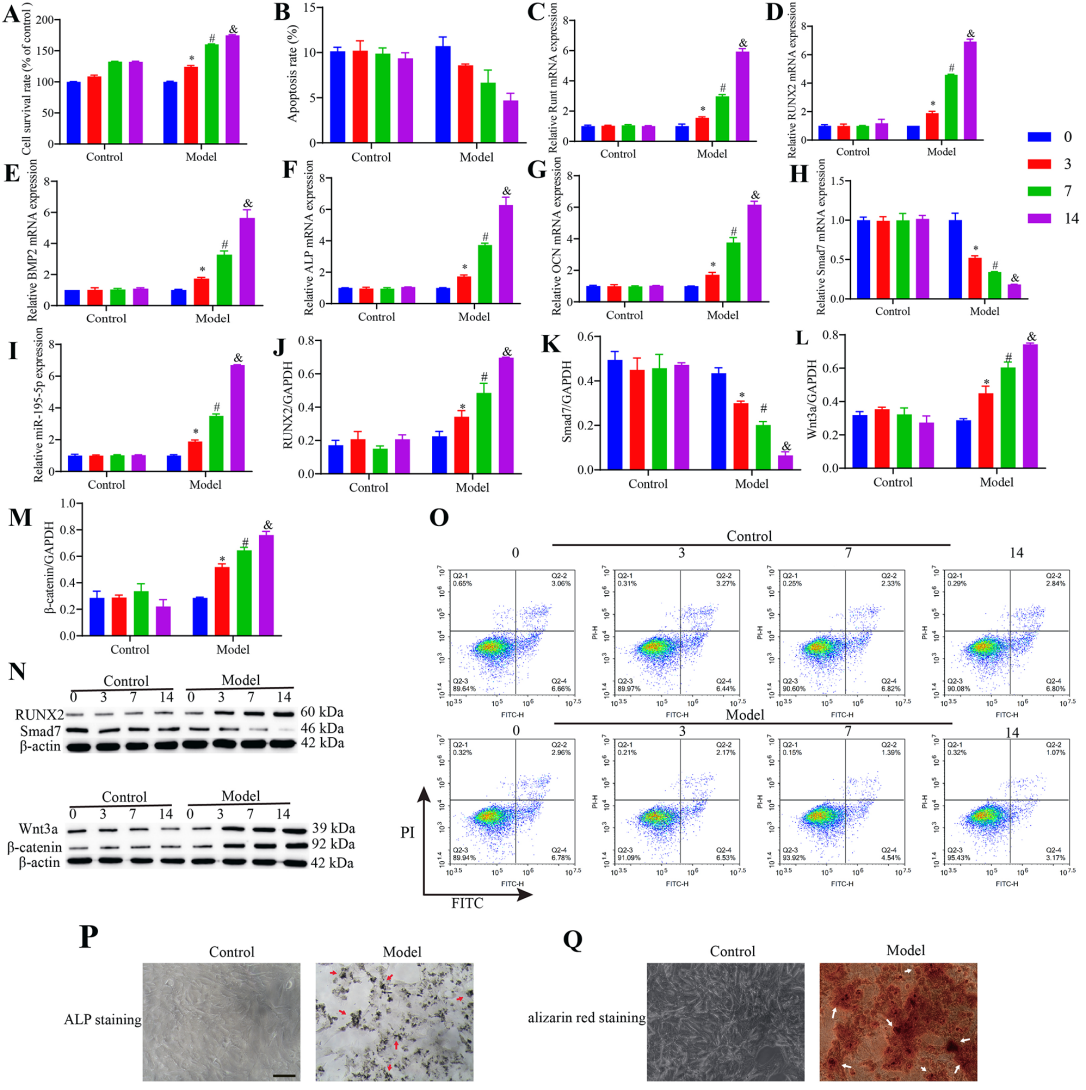
miR-195-5p promotes the osteogenic differentiation of vascular smooth muscle cells (VSMCs) via the Wnt/β-catenin pathway. (**A**) Cell viability was determined using an MTT assay. (**B**, **O**) Cell apoptosis was determined using flow cytometry. (**C**–**I**) The levels of Runt, RUNX2, BMP2, ALP, OCN, and Smad7 mRNAs and miR-195-5p were determined using RT-qPCR. (**J**–**N**) The levels of RUNX2, Smad7, Wnt3a, and β-catenin proteins were determined using western blotting. (**P**) Alkaline phosphate staining. (Q) Alizarin Red staining (×200). Scale bar, 1000 μm, *p < 0.05 vs. 0 h group, #*p* < 0.05 vs. 3 h group, and *p* < 0.05 vs. 7 h group

As shown in Fig. 1, osteogenic differentiation of VSMCs was induced by β-GP at 3, 7, and 14 days, and at each of these time points, cell survival, mRNA levels of Runt, RUNX2, BMP2, ALP, OCN, and miR-195-5p, and protein levels of RUNX2, Wnt3a, and β-catenin were found to be higher in the model group than in the control group. In contrast, the levels of cell apoptosis and Smad7 mRNA and protein were lower in the model group than in the control group.

In the model group, we detected gradual increases in cell survival, the mRNA levels of Runt, RUNX2, BMP2, ALP, OCN, and miR-195-5p, and the protein levels of RUNX2, Wnt3a, and β-catenin with an increase in β-GP intervention time, whereas the levels of cell apoptosis and Smad7 mRNA were characterized by a gradual decline. Compared with the control group cells, we observed more extensive ALP and alizarin Red staining of model group VSMCs, thereby providing evidence to indicate the osteogenic differentiation of these cells.

### miR-195-5p promotes the osteogenic differentiation of VSMCs and expression of Wnt/β-catenin pathway components

**Fig. 2:**
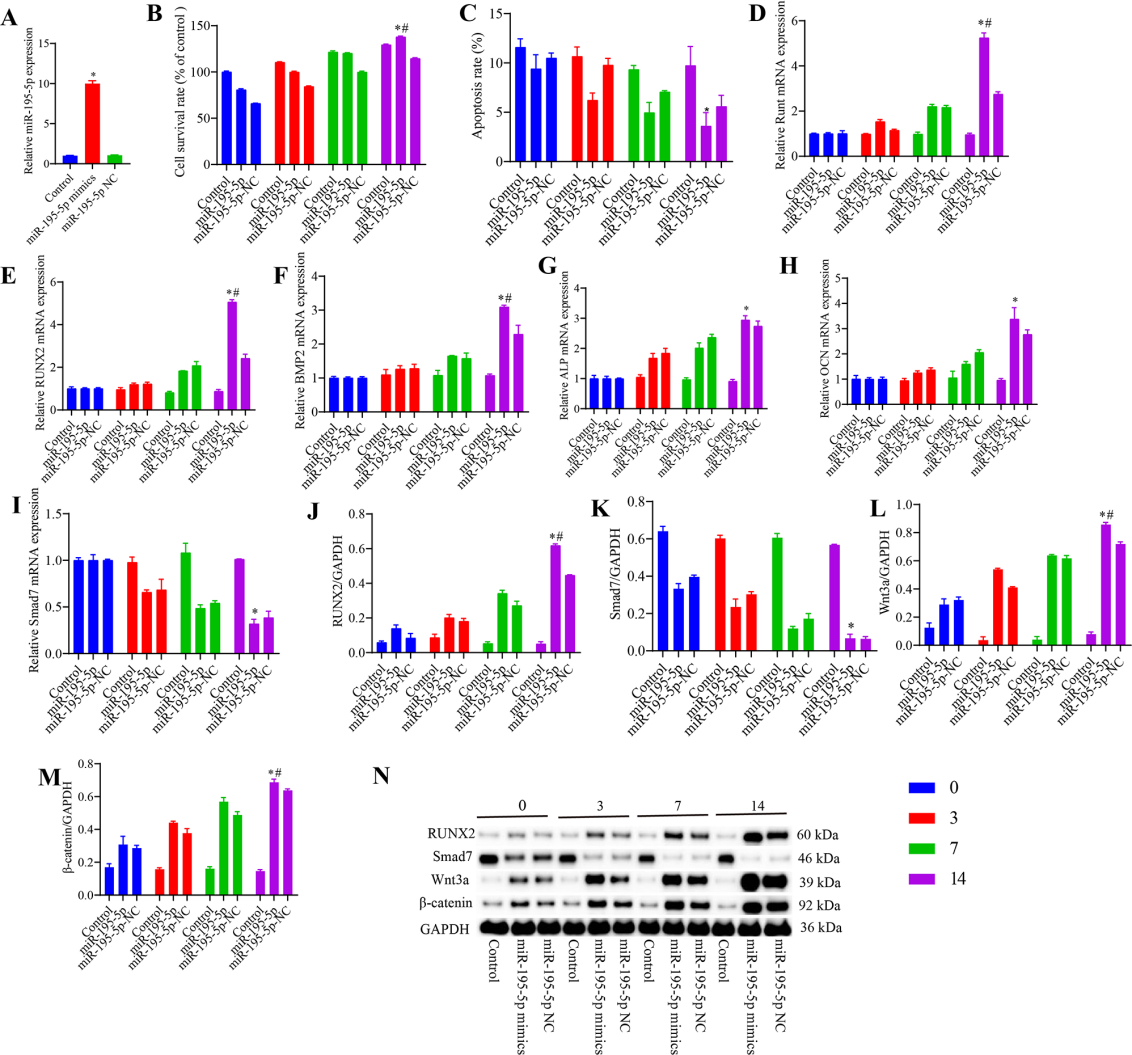
The effects of miR-195-5p on the osteogenic differentiation of vascular smooth muscle cells (VSMCs) and expression of Wnt/β-catenin pathway constituents. (**A**) Levels of miR-195-5p were determined using RT-qPCR. (**B**) Cell viability was determined using an MTT assay. (**C**) Cell apoptosis was determined using flow cytometry. (**D**–**I**) The levels of Runt, RUNX2, BMP2, ALP, OCN, and Smad7 mRNAs were determined using RT-qPCR. (**J**–**N**) The levels of RUNX2, Smad7, Wnt3a, and β-catenin proteins were determined using western blotting. *p < 0.05 vs. control group; #*p* < 0.05 vs. miR-195-5p-NC group

**Fig. 3:**
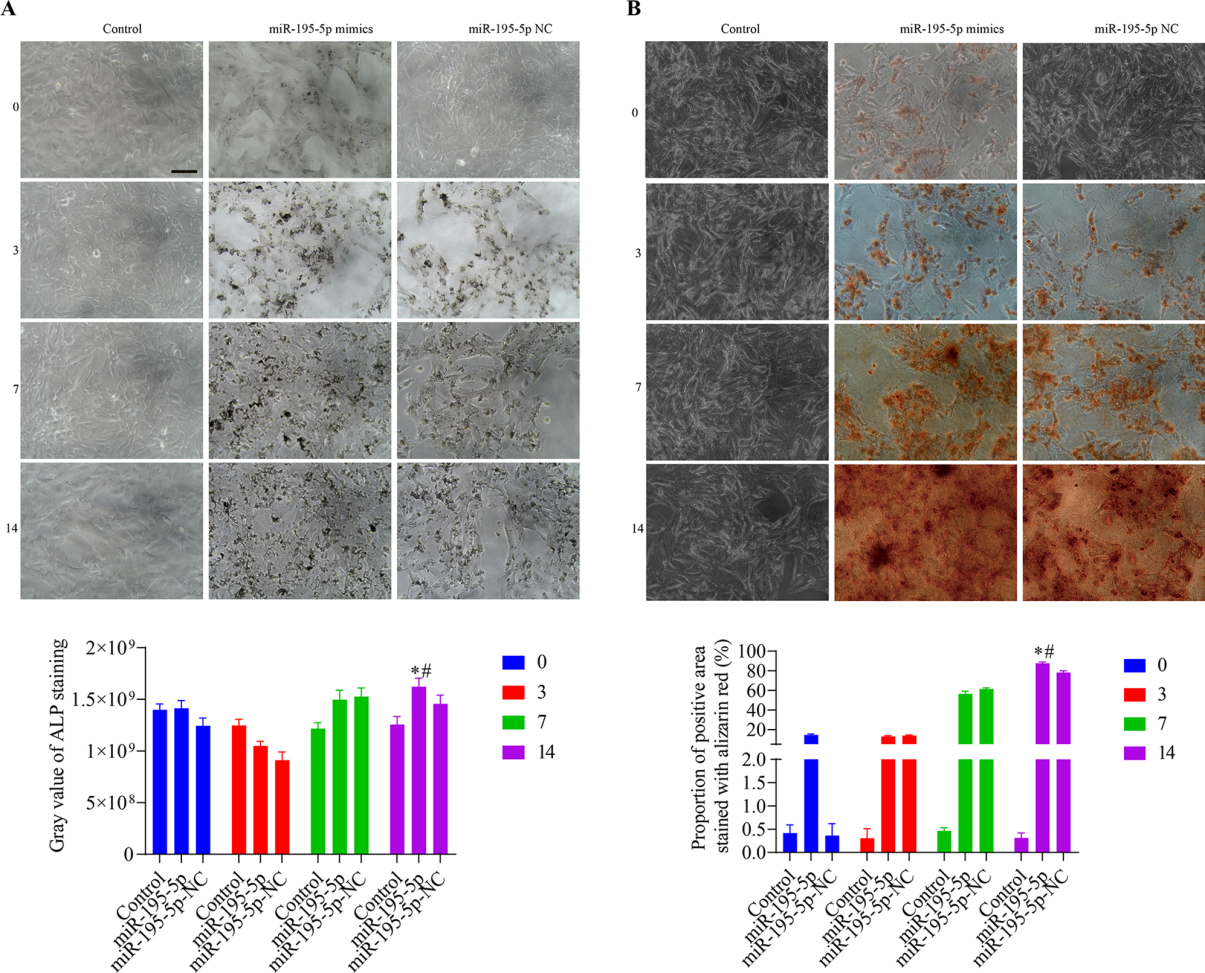
Staining. (**A**) Alkaline phosphatase staining. (**B**) Alizarin Red staining. Scale bar, 1000 μm. Control group: vascular smooth muscle cells (VSMCs) without osteogenic differentiation; miR-195-5p mimic group: induction of VSMC osteogenesis after transfection with an miR-195-5p mimic sequence. miR-195-5p NC group: induction of VSMC osteogenesis following transfection with an miR-195-5p negative control sequence

In contrast to VSMCs in the control group, which did not undergo osteogenesis, VSMCs in the miR-195-5p NC group transfected with miR-195-5p NC sequences were found to undergo differentiation via osteogenesis, as were VSMCs in the miR-195-5p group transfected with miR-195-5p mimic sequences. Following osteogenic differentiation, we detected an upregulation of miR-195-5p in VSMCs, which gradually increased with an increase in β-GP intervention time. However, we found that miR-195-5p influences VSMC viability and osteogenic differentiation, although the effects on the Wnt/β-catenin pathway remained undetermined. Thus, miR-195-5p levels in VSMCs increased by transfection of miR-195-5p-mimics by liposomes, evaluating osteogenic differentiation and changes in the Wnt/β-catenin pathway. Similarly, following the transfection of VSMCs with miR-195-5p-mimics/NC and induction with β-GP for 3, 7, and 14 days, we found that compared with the control group cells, there were significant increases in the levels of miR-195-5p in the miR-195-5p-mimic group cells, thereby confirming the successful transfection with miR-195-5p. In these latter cells, we detected gradual increases in cell survival, the levels of Runt, RUNX2, BMP2, ALP, and OCN mRNAs, and the levels of RUNX2, Wnt3a, and β-catenin proteins with an increase in β-GP intervention time (Fig. 2), whereas there were gradual reductions in the levels of cell apoptosis (Fig. S1) and Smad7 levels. When assessed on day 14 of intervention, we found that compared with the control group cells, there were significant increases in cell survival, the levels of Runt, RUNX2, BMP2, ALP, and OCN mRNAs, and the levels of RUNX2, Wnt3a, and β-catenin proteins in cells of the miR-195-5p-mimic group, whereas significant reduction were detected in cell apoptosis and the mRNA and protein levels of Smad7. In addition, compared with the miR-195-5p-mimics group, we observed less extensive ALP and alizarin Red staining of VSMCs in the miR-195-5p-NC group cells (Fig. 3).

### Effects of Smad7 on the osteogenic differentiation of VSMCs and the Wnt/β-catenin pathway

**Fig. 4:**
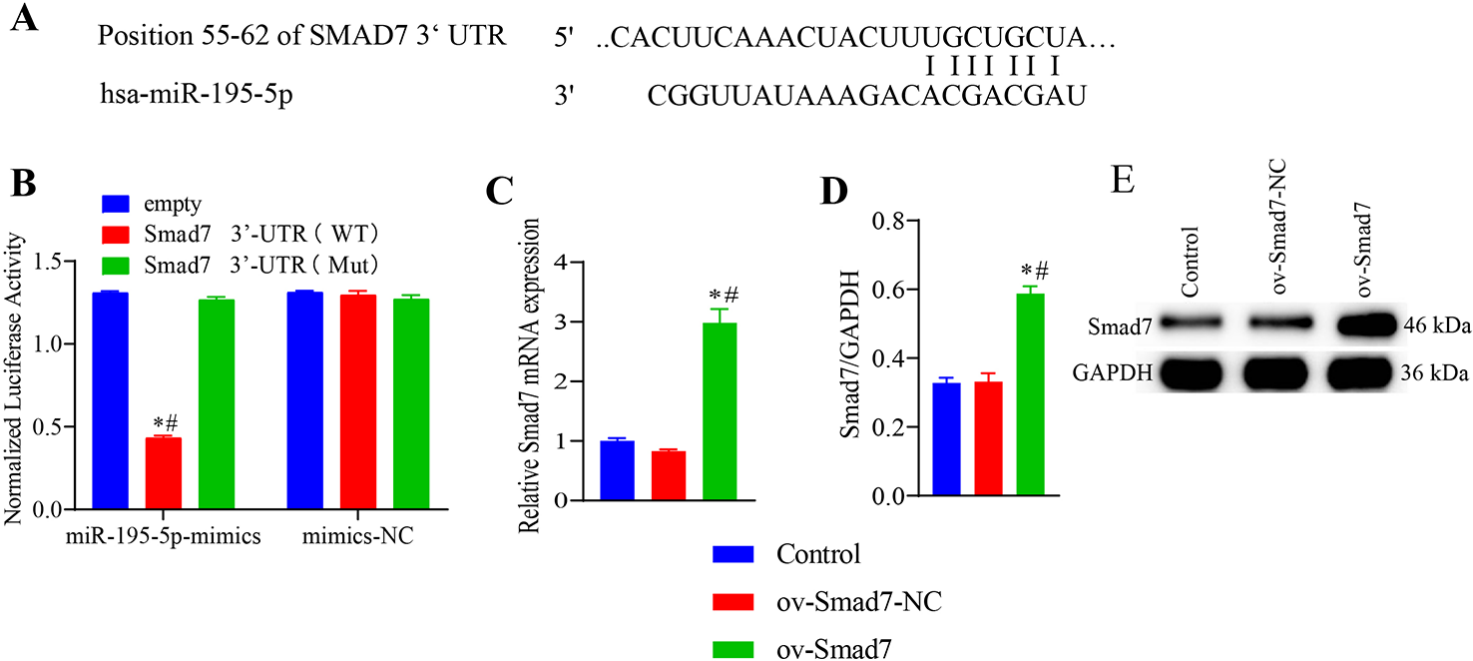
Dual-luciferase reporter assay and transfection. (**A**) An alignment of Smad7 mRNA 3ʹUTR with the miR-195-5p seed sequence. (**B**) Dual-luciferase reporter assay. *p < 0.05 vs. empty vector group, #*p* < 0.05 vs. Smad7 3′-UTR (Mutant) group. (**C**) The levels of Smad7 mRNA were determined using RT-qPCR. (**D** and **E**) The levels of Smad7 protein were determined using western blotting. *p < 0.05 vs. control group, #*p* < 0.05 vs. ov-Smad7-NC group

**Fig. 5:**
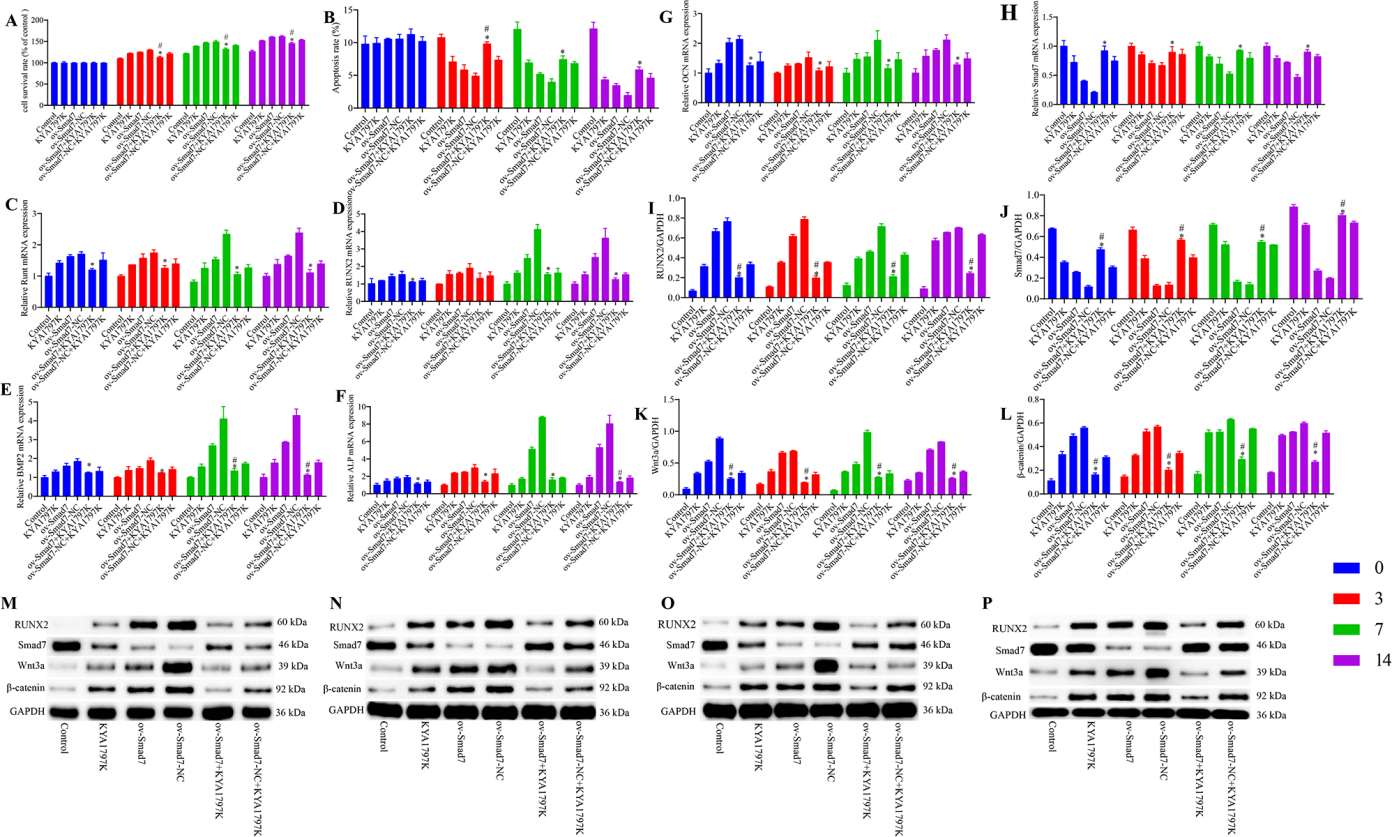
The effects of Smad7 on the osteogenic differentiation of vascular smooth muscle cells (VSMCs) and the Wnt/β-catenin pathway. (**A**) Cell viability was determined using an MTT assay. (**B**) Cell apoptosis was determined using flow cytometry. (**C**–**H**) The levels of Runt, RUNX2, BMP2, ALP, OCN, and Smad7 mRNAs were determined using RT-qPCR. (**I**–**P**) The levels of RUNX2, Smad7, Wnt3a, and β-catenin proteins were determined using western blotting. *p < 0.05 vs. ov-Smad7 group, #*p* < 0.05 vs. ov-Smad7-NC + KYA1797K group. M, N, O, and P are blots obtained for days 0, 3, 7, and 14 of osteogenic differentiation, respectively. Control group: VSMCs without osteogenic differentiation. Other groups: VSMCs with osteogenic differentiation

**Fig. 6:**
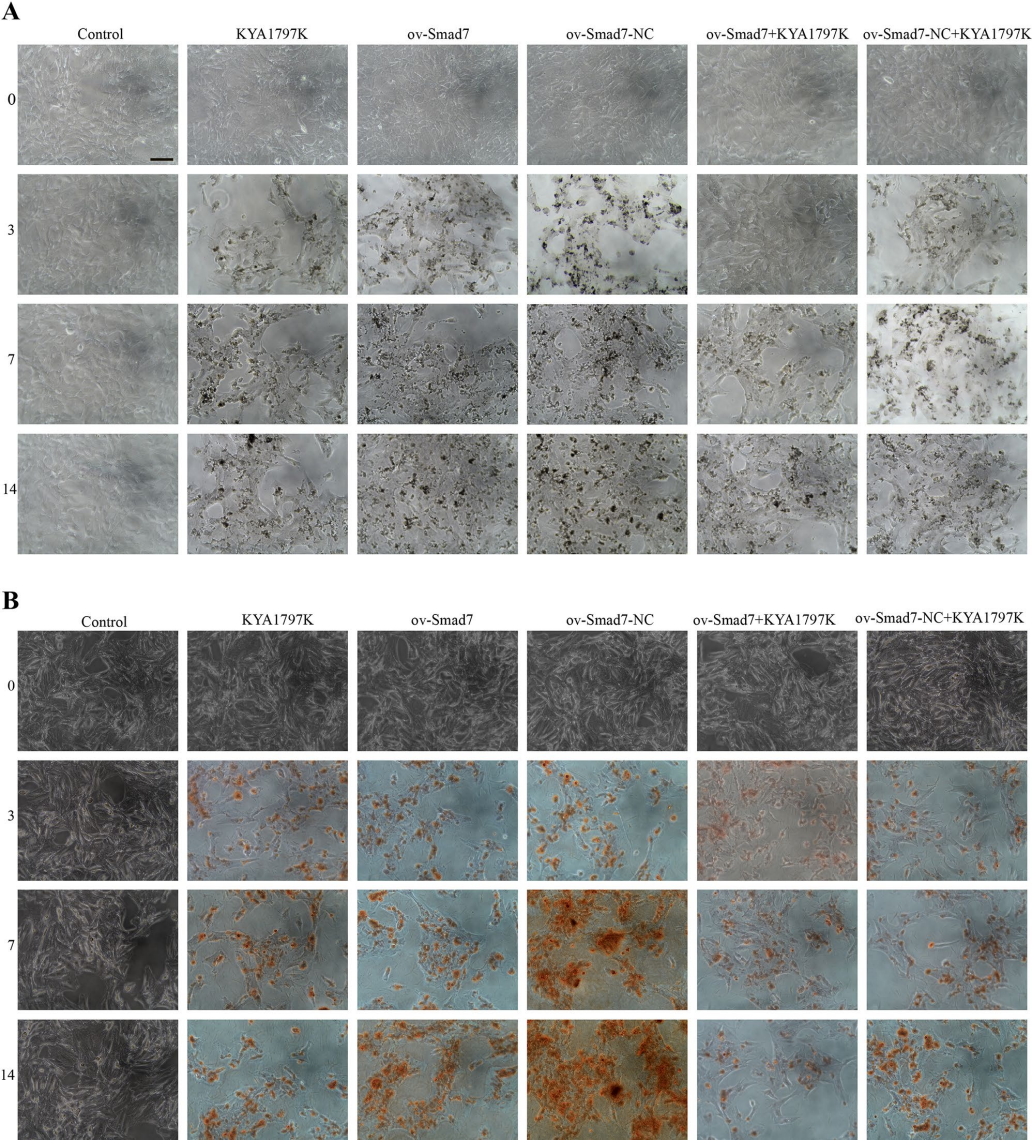
Staining. (**A**) Alkaline phosphatase staining. (**B**) Alizarin Red staining. Scale bar, 1000 μm. Control group: vascular smooth muscle cells (VSMCs) without osteogenic differentiation. Other groups: VSMCs with osteogenic differentiation

Both osteogenic differentiation and miR-195-5p were found to be associated with a reduction in Smad7 levels in VSMCs. We speculate that Smad7 mediates the osteogenic differentiation via miR-195-5p and the Wnt/β-catenin pathway. Figure 4A shows a comparison of Smad7 mRNA 3ʹUTR and miR-195-5p seed sequences. In the dual-luciferase reporter experiment, we found that compared with the empty vector and Smad7 3′-UTR (Mut) groups, there was a significant reduction in normalized luciferase activity in the Smad7 3′-UTR (WT) group following transfection with miR-195-5p-mimics (Fig. 4B), thereby indicating that miR-195-5p interacts with Smad7. Accordingly, we subsequently transfected VSMCs with an Smad7 overexpression vector (ov-Smad7), and found that compared with the control and ov-Smad7-NC groups, there were significant increases in the mRNA and protein expression levels in the ov-Smad7 group (Fig. 4C–E). Thereafter, we examined the effects of Smad7 on the osteogenic differentiation of VSMCs and the Wnt/β-catenin pathway. VSMCs were treated with 25 µM Wnt pathway inhibitor (KYA1797K) and transfected with ov-Smad7, and β-GP was used for induction for 3, 7, and 14 days. After 3 days of induction, we found that compared with the control group, there were increases in the cell survival, levels of Runt, RUNX2, BMP2, ALP, and OCN mRNAs, and levels of RUNX2, Wnt3a, and β-catenin proteins in the ov-Smad7 group cells (Fig. 5), whereas there were corresponding reduction in cell apoptosis (Fig. S2), and the mRNA and protein levels of Smad7 in the ov-Smad7 group cells. Compared with cells in the ov-Smad7 group, on days 3, 7, and 14 of intervention, we detected significant increases in cell survival, levels of Runt, RUNX2, BMP2, ALP, and OCN mRNAs, and levels of RUNX2, Wnt3a, and β-catenin proteins in the ov-Smad7 + KYA1797K group cells, whereas significant reduction were observed with respect to cell apoptosis and the mRNA and protein levels of Smad7. Furthermore, ALP and alizarin Red staining (Fig. 6) revealed reductions in the expression of ALP and the number of mineralized nodules among the ov-Smad7 group cells compared with those in the ov-Smad7-NC group. Similar reductions in ALP expression and the number of mineralized nodules were observed in response to treatment with KYA1797K. Moreover, we established that compared with the control group cells, there was a more pronounced osteogenic differentiation of cells in the ov-Smad7 and ov-Smad7-NC groups (Fig. 5). However, compared with cells in the ov-Smad7-NC + KYA1797K group, there were lower levels of Runt, RUNX2, BMP2, ALP, and OCN mRNAs in the ov-Smad7 + KYA1797K group cells. Collectively, these findings indicate that an overexpression of Smad7 can inhibit the β-GP-induced osteogenic differentiation of VSMCs, which we suspect could be associated with the activity of the Wnt/β-catenin pathway.

## Discussion

Recently, miRNAs, which regulate gene expression at the RNA level and play roles in multiple cellular processes, including cell growth, division, signaling, and development, have been the focus of extensive research. miRNAs also play important roles in tumor initiation and development [23]. Among these multifarious effects, the findings of several studies have indicated that miRNAs are involved in the osteogenic differentiation of VSMCs. For example, it has been established that by promoting the expression of miR-145, vitamin D attenuates the uremia-induced osteogenic differentiation of the aorta [24], whereas miRNA-223-3p has been demonstrated to inhibit the osteogenic switch of VSMCs [25]. Notably, whereas a few miRNAs have been found to play an inhibitory role in osteogenic differentiation, others promote this process [26]. Currently, however, there have been few studies that have examined the associations between miR-195-5p and osteoblastic differentiation, although among those studies that have been conducted, the overexpression of miR-195-5p has been found to inhibit osteogenic differentiation and bone formation in human periodontal ligament cells [27].

In the present study, we demonstrated that β-GP induces the osteogenic differentiation of VSMC, which in turn promotes cell proliferation and reduces cell apoptosis. The findings of previous studies, based on the detection of osteogenic differentiation genes at the transcriptional and translational levels using ALP and Alizarin Red staining, have indicated that VSMCs promote osteogenic differentiation. In this study, we obtained evidence to indicate the regulation of miR-195-5p, Smad7, Wnt3a, and β-catenin during osteogenic differentiation. Comparisons between the control and model group cells during the period of osteogenic induction by β-GP revealed increases in the expression of miR-195-5p, Wnt3a, and β-catenin over time and a corresponding reduction in the expression of Smad7. The findings of a recent study have similarly indicated that carbon monoxide-releasing molecule 3 promotes osteogenic differentiation of rat BMSCs via miR-195-5p/Wnt3a [28], whereas other studies have confirmed that miR-195-5p enhances the osteogenic differentiation of cells and is associated with Smad7 [29, 30]. These findings accordingly persuaded us to focus our research on the effects of miR-195-5p and Smad7 on the osteogenic differentiation of VSMCs and the Wnt/β-catenin pathway.

Smad7 has been identified as a nuclear protein that negatively regulates the transforming growth factor-β (TGF-β) signaling pathway [31]. Zhang et al. have reported that miR-195-5p targets Smad7 to regulate phenotypic switching in cavernous smooth muscle cells [32], whereas Chen et al. established that bone marrow mesenchymal stem cell exosomal miR-195-5p targets the TGF-β/Smad7 pathway [33], and Ding et al. demonstrated that by targeting Smad7, miR-195-5p contributes to inhibiting the TGF-β pathway [34]. Similarly, the findings of our dual-luciferase reporter assays in the present study provided evidence to indicate that miR-195-5p targets Smad7, thereby resulting in reductions of this factor at both the mRNA and protein levels. Although the targeting of Smad7 by miR-195-5p has previously been established, to the best of our knowledge, this study is the first to reveal the Smad7-targeting role of miR-195-5p in the context of the osteogenic differentiation of VSMCs. Specifically, our findings indicate that by regulating Smad7, miR-195-5p promotes the osteogenic differentiation of VSMCs. Moreover, by inhibiting the expression of Smad7, miR-195-5p promotes activation of the Wnt/β-catenin pathway, which is closely associated with osteogenic differentiation. Numerous studies have reported the involvement of the Wnt/β-catenin pathway in osteogenic differentiation [35–37], which we confirmed in the present study with respect to the osteogenic differentiation of VSMCs. However, despite demonstrating that miR-195-5p inhibits Smad7 and activates the Wnt/β-catenin pathway and osteogenic differentiation of VSMCs, the role of Smad7 in the miR-195-5p/Smad7/Wnt/β-catenin pathway axis has yet to be sufficiently established.

Zhou et al. have reported that targeting Smad7 similarly promotes the osteogenic differentiation of BMSCs [38], and that following KYA1797K intervention, the overexpression of Smad7 does not promote increases in the levels of Runt, RUNX2, BMP2, ALP, OCN, Wnt3a, or β-catenin. Han et al. found that Smad7 regulates osteogenic differentiation during osteonecrosis of the femoral head [39], and Wei et al. have established that Smad7 plays an essential role in nuclear accumulation in the Wnt/β-catenin pathway [40]. To gain a better understanding of the role of Smad7 in the miR-195-5p-mediated activation of Wnt/β-catenin signaling and osteogenic differentiation, we transfected VSMCs with an ov-Smad7 vector, and simultaneously treated the transfected cells with the Wnt/β-catenin pathway inhibitor KYA1797K. Subsequent assessments of osteogenic differentiation capacity and the levels of Wnt3a and β-catenin revealed that overexpression of Smad7 promotes the osteogenic differentiation of VSMCs and upregulates the expression of Wnt3a and β-catenin at the mRNA and protein levels.

In this study, we sought to elucidate the mechanisms of action of miR-195-5p with respect to the osteogenic differentiation of VSMCs, and accordingly observed that by targeting Smad7, miR-195-5p promotes the Wnt/β-catenin pathway and osteogenic differentiation of VSMCs. Furthermore, given that VSMCs occur within in calcified arterial media, we assume that the osteogenic differentiation and calcification of VSMCs are associated with the progression of AMC. Collectively, our findings thus provide evidence indicating that miR-195-5p targets Smad7 and activates the Wnt/β-catenin pathway, thereby regulating the osteogenic differentiation and calcification of VSMCs. These findings provide novel insights and will potentially serve as a reference for the targeted therapy of AMC.

## Conclusion

**Fig. 7:**
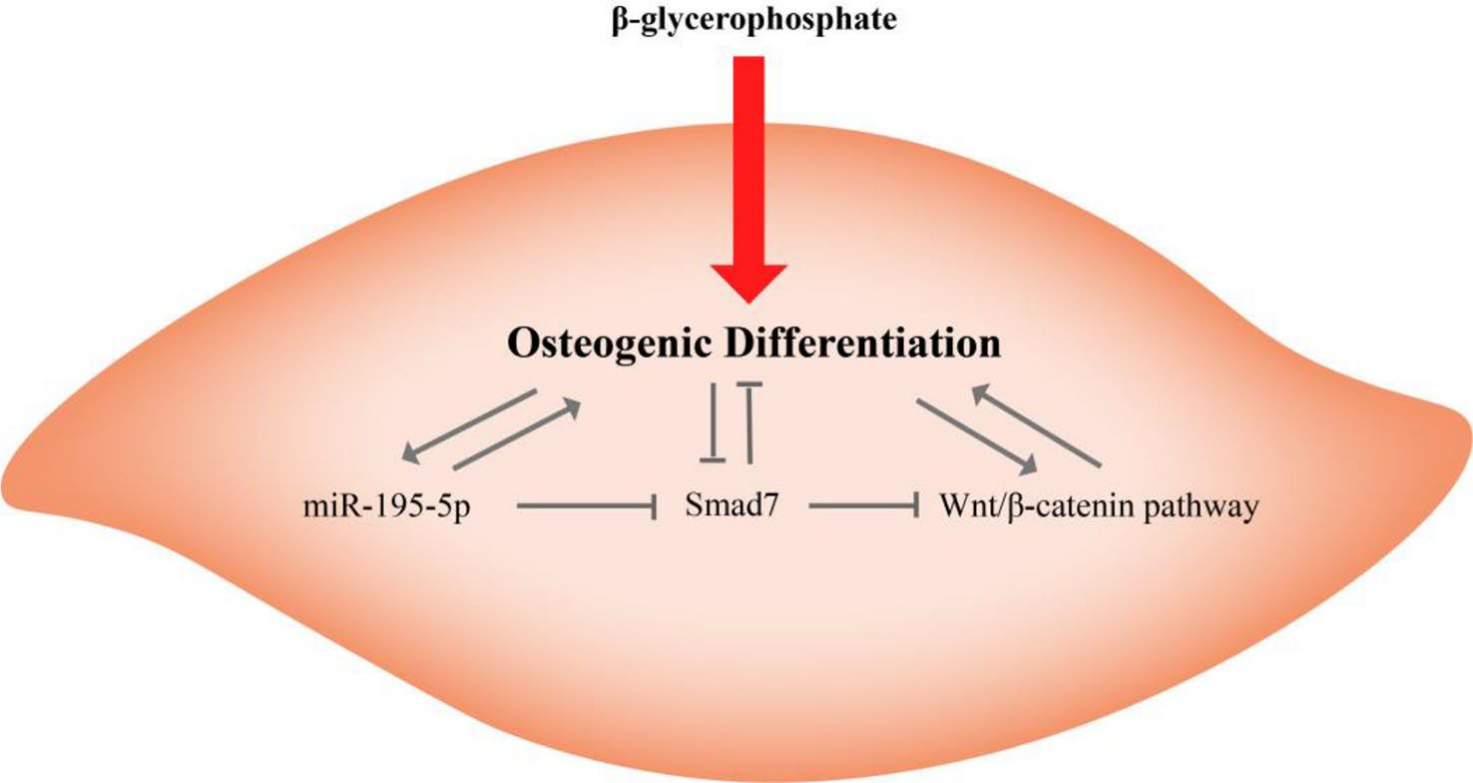
Graphical summary

By inhibiting the expression of Smad7, miR-195-5p activates the Wnt/β-catenin pathway and thereby promotes the osteogenic differentiation of VSMCs. In the absence of this negative regulatory activity, Smad7 inhibits the Wnt/β-catenin pathway and osteogenic differentiation (Fig. 7). Despite these important findings, this study does have certain limitations, notably the fact that we did not conduct any complementary animal experiments. Accordingly, in further studies, we will validate the in vitro findings of this study by performing in vivo experiments and collecting clinical data, thereby providing a valuable reference for the field.

### Electronic supplementary material

Below is the link to the electronic supplementary material.

Supplementary Material 1

Supplementary Material 2

Supplementary Material 3

Supplementary Material 4

## Data Availability

The datasets used and/or analyzed during the this study are available from the corresponding author on reasonable request.

## Abbreviations

AMC: arterial media calcification
VSMCs: vascular smooth muscle cells
miRNAs: microRNAs
β-GP: β-glycerophosphate
MTT: 3-(4,5-dimethylthiazol-2-yl)-2,5-diphenyltetrazolium bromide
WT: wild type
MT: mutant
