# Supplementary material for: Mir-195-5p targets Smad7 regulation of the Wnt/β-catenin pathway to promote osteogenic differentiation of vascular smooth muscle cells
Source: BMC Cardiovasc Disord. 2024 Apr 23;24:221. doi: 10.1186/s12872-024-03891-2 (PMC11036659; doi:10.1186/s12872-024-03891-2)

figure 1N

RUNX2

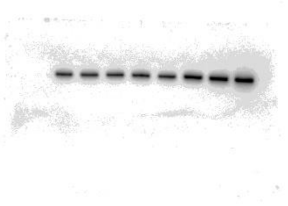

Smad

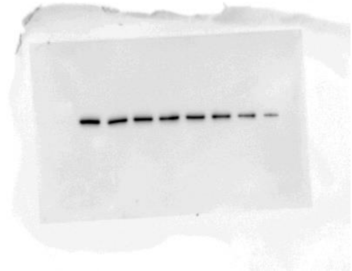

WNT3A

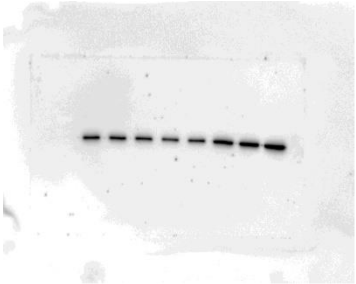

β -actin(1)

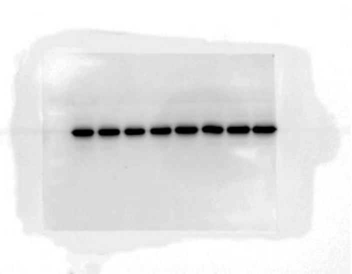

β -actin

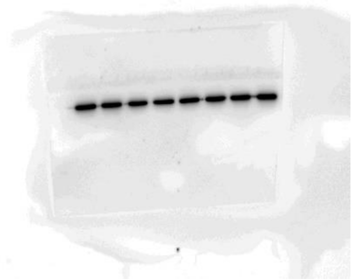

β-Catein(2)

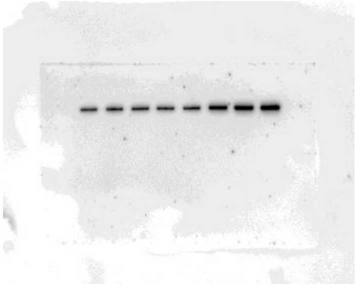

figure 2N

Runx2

Smad7

Wnt3a

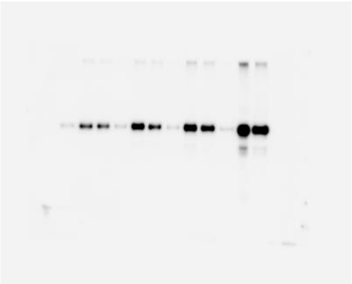

β-catenin

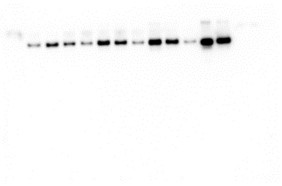

GAPDH

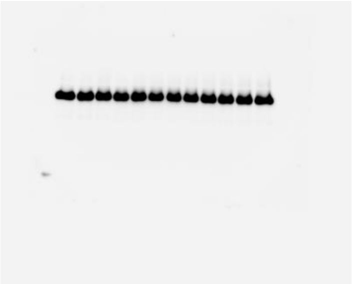

figure 4E

Smad7

GAPDH

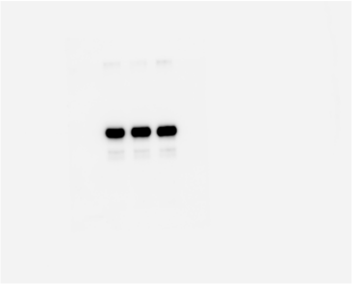

figure 5M

RUNX2

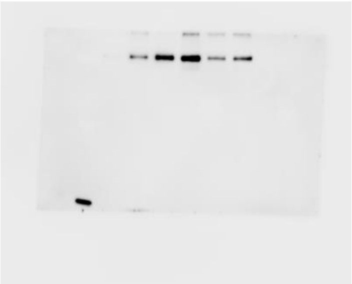

smad7

Wnt3a

β -Catenin

GAPDH

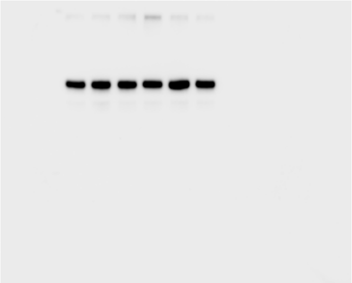

figure 5N

RUNX2

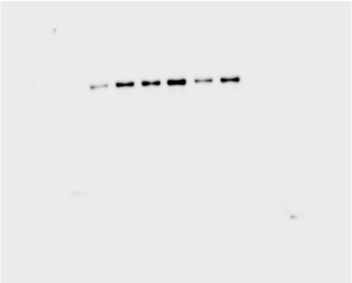

SMAD7

Wnt3a

β -Catenin

GAPDH

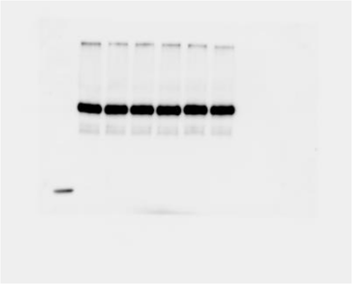

figure 5O

RUNX2

Smad7

Wnt3a

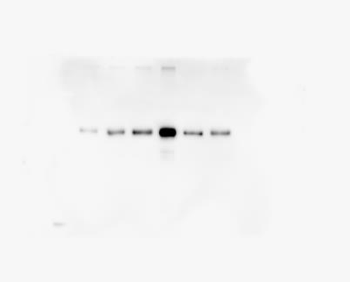

β -Catenin-1

GAPDH

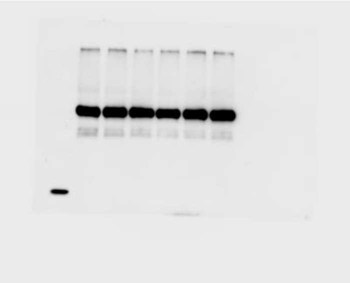

figure 5P

RUNX2

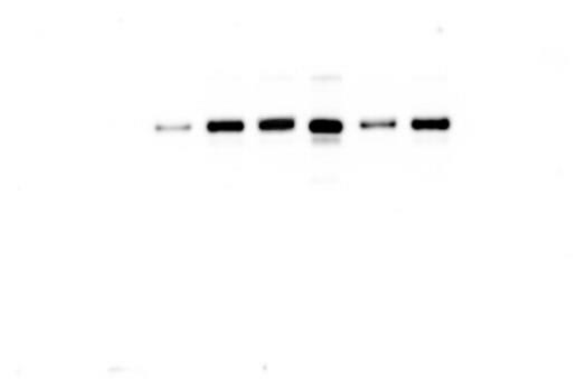

Smad7

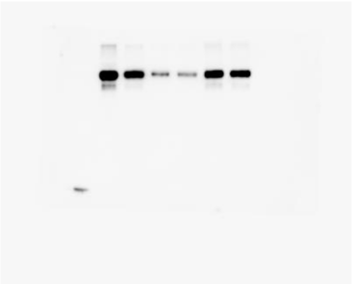

Wnt3a

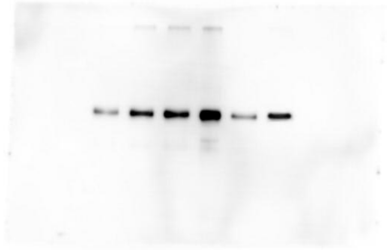

β -catenin

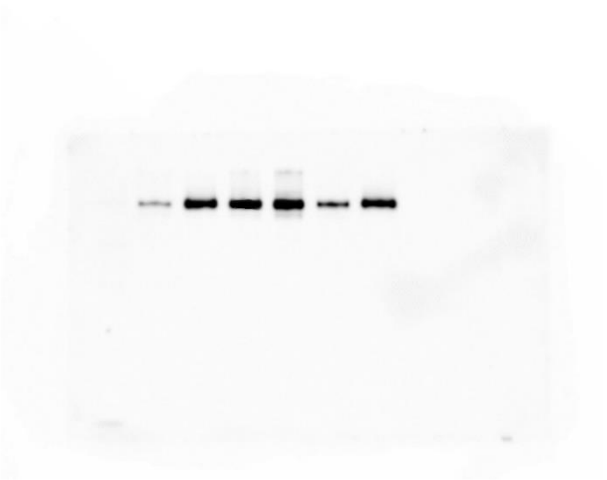

GAPDH

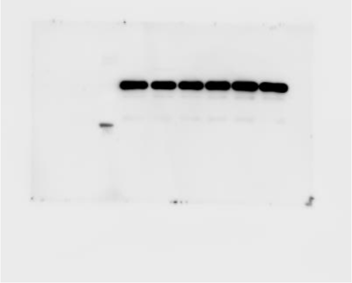

Supplement: Supplementary file 2 — Supplementary Material 2 [file 12872_2024_3891_MOESM2_ESM.pdf]
