# Supplementary figures and images for: Mir-195-5p targets Smad7 regulation of the Wnt/β-catenin pathway to promote osteogenic differentiation of vascular smooth muscle cells
Source: BMC Cardiovasc Disord. 2024 Apr 23;24:221. doi: 10.1186/s12872-024-03891-2 (PMC11036659; doi:10.1186/s12872-024-03891-2)

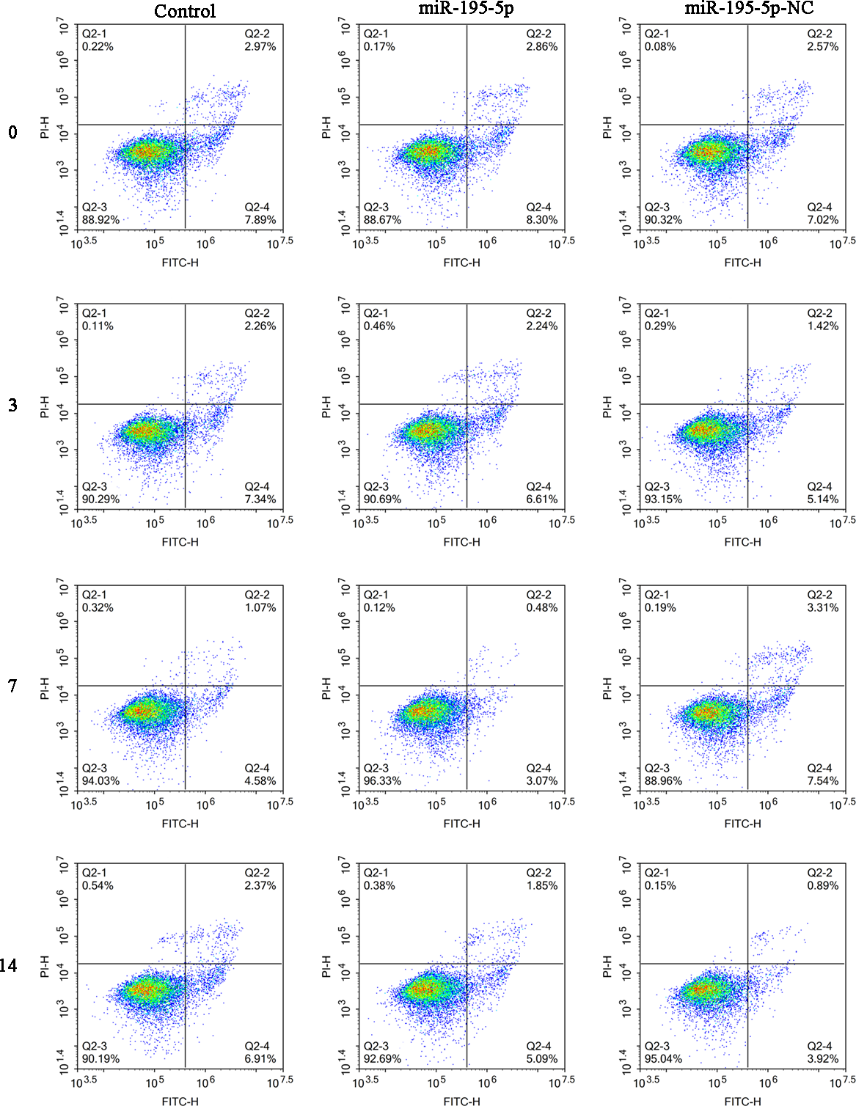


figure s1 Flow cytometry was used to detect cell apoptosis.

Supplement: Supplementary file 3 — Supplementary Material 3 [file 12872_2024_3891_MOESM3_ESM.docx]

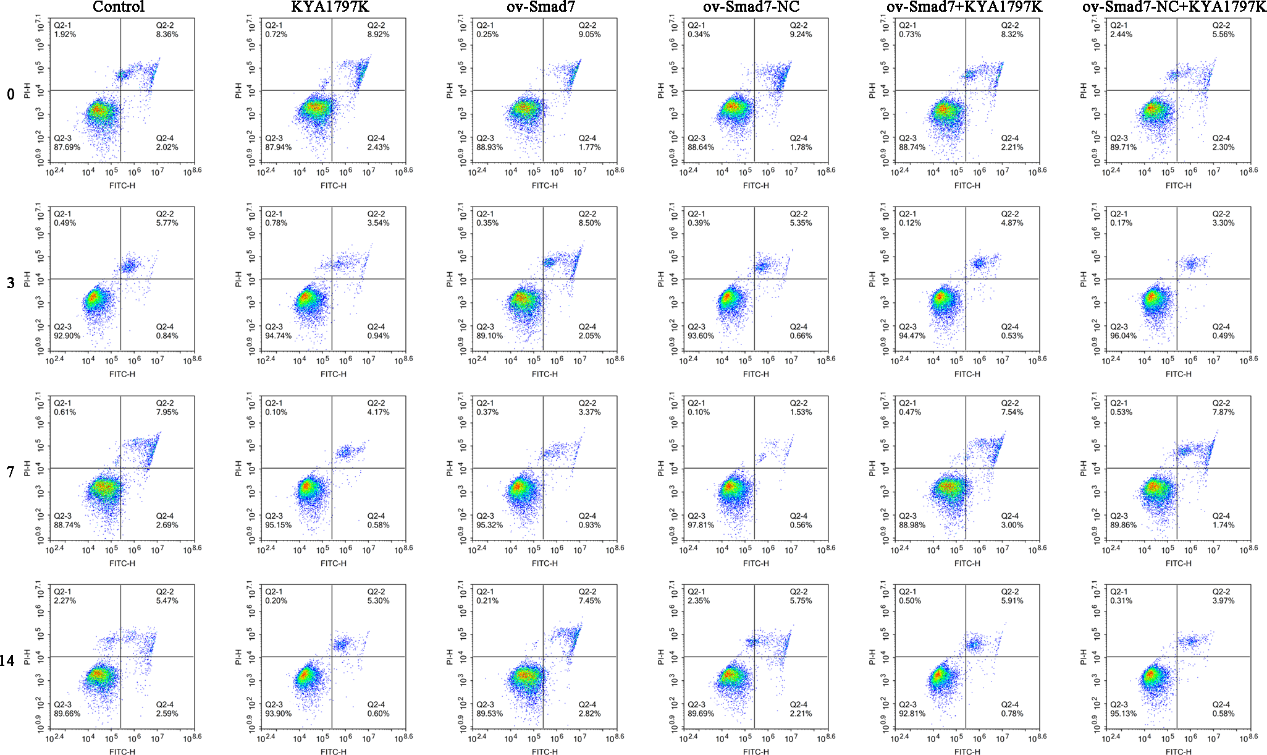


figure s2 Flow cytometry was used to detect cell apoptosis.

Supplement: Supplementary file 4 — Supplementary Material 4 [file 12872_2024_3891_MOESM4_ESM.docx]
